# Genotypic Variation for Salinity Tolerance in *Cenchrus ciliaris* L.

**DOI:** 10.3389/fpls.2016.01090

**Published:** 2016-07-28

**Authors:** Abdullah J. Al-Dakheel, M. Iftikhar Hussain

**Affiliations:** Crop Diversification and Genetic Improvement Section, International Center for Biosaline AgricultureDubai, United Arab Emirates

**Keywords:** buffelgrass, biomass yield, multivariate analysis, salt tolerance, genotypic diversity

## Abstract

Scarcity of irrigation water and increasing soil salinization has threatened the sustainability of forage production in arid and semi-arid region around the globe. Introduction of salt-tolerant perennial species is a promising alternative to overcome forage deficit to meet future livestock needs in salt-affected areas. This study presents the results of a salinity tolerance screening trial which was carried out in plastic pots buried in the open field for 160 buffelgrass (*Cenchrus ciliaris* L.) accessions for three consecutive years (2003–2005). The plastic pots were filled with sand, organic, and peat moss mix and were irrigated with four different quality water (EC 0, 10, 15, and 20 dS m^−1^). The results indicate that the average annual dry weights (DW) were in the range from 122.5 to 148.9 g/pot in control; 96.4–133.8 g/pot at 10 dS m^−1^; 65.6–80.4 g/pot at 15 dS m^−1^, and 55.4–65.6 g/pot at 20 dS m^−1^. The highest DW (148.9 g/pot) was found with accession 49 and the lowest with accession 23. Principle component analysis shows that PC-1 contributed 81.8% of the total variability, while PC-2 depicted 11.7% of the total variation among *C. ciliaris* accessions for DW. Hierarchical cluster analysis revealed that a number of accessions collected from diverse regions could be grouped into a single cluster. Accessions 3, 133, 159, 30, 23, 142, 141, 95, 49, 129, 124, and 127 were stable, salt tolerant, and produced good dry biomass yield. These accessions demonstrate sufficient salinity tolerance potential for promotion in marginal lands to enhance farm productivity and reduce rural poverty.

## Introduction

Salt-affected soils and millions of hectares of marginal lands have limited the scope for crop production (Wang et al., 2012). According to the Food and Agriculture Organization (FAO), 34 million hectares (11% of the total irrigated area of the world) are affected by different levels of salinization (Food Agriculture Organization of the United Nations, 2012). Moreover, the world loses 0.25–0.5 M ha of agricultural land annually because of salt buildup, which mainly results from irrigation, especially in arid and semiarid areas (Peng et al., 2008; Qadir et al., 2014). Soil salinity reduces the productivity of most crops, although to a varying extent depending on species (Roy et al., 2014; Hussain et al., 2016). Besides improving water management practices to reduce salt accumulation in the root zone, there is a need to improve salinity tolerance of strategically important crops. The use of low quality saline water for plant production is an option to conserve limited freshwater resources, particularly for the water-scarce regions of Arabian and African peninsula.

The cultivation of forages, biomass crops, and perennial grasses as feedstock for energy, biomaterials, and livestock rearing have been promoted as an opportunity to improve sustainability of forage supply, energy security, and contributing to the rural development (Ahmad, 2010). Therefore, demand for sustainable biomass production for livestock and energy use has raised the interest in perennial crops like *Cenchrus ciliaris* L. Buffelgrass (*C. ciliaris* L.) is a perennial (C_4_) forage grass (family poaceae), sometimes produces rhizomes and is native to the Arabian Peninsula. The *C. ciliaris* is dominant in natural grazing zones of Ethiopia (Angassa and Baars, 2000), Australia (Buldgen and Francois, 1998), and North Africa (Mseddi et al., 2004). Buffelgrass has proved useful for pasture and soil retention in a wide range of environments due to its drought tolerance, high biomass, deep roots, rapid response to summer rains, and resistance to overgrazing. With extensive belowground systems, cultivation of perennial grasses present high efficiencies in the use of nutrient and water resources and control of soil erosion, carbon sequestration with the restoration of soil properties (fertility, structure, organic matter). Compared with annual systems, herbaceous perennial crops have the advantages of erodibility, and crop management options, such as pesticides and fertilizers inputs (Zhang et al., 2011; Fernando et al., 2012). The salt tolerance of different *C. ciliaris* genotypes need to be evaluated to test their suitability for marginal environments to offer a more practical solution for effective utilization of salt affected soils. Among buffelgrass, accession from North America, “Texas 4464” has been reported as drought tolerant (Ayerza, 1981), and “Biloela” as salt-tolerant (Graham and Humphreys, 1970). Therefore, strategies for mitigating salinity problems in crop production include both development of management options (Shannon, 1997) and genetic improvement of current cultivars (Krishnamurthy et al., 2007).

Germplasm of a specific crop collected from the diverse sources offers greater genetic diversity and may furnish useful traits to widen the genetic base of crop species. The collection, screening and description of the existing variability among the forage crops are the first step in the performance evaluation and selection process (Ponsens et al., 2010). Knowledge about germ- plasm diversity and salinity tolerance evaluation will be an excellent tool to screen and select high yielding accessions for further evaluation under field conditions. Screening large numbers of genotypes for salinity tolerance in the field is notoriously difficult because of the variability of salinity within fields (Daniells et al., 2001). Moreover, it would be difficult to determine the critical parameters under field conditions since any environmental change could result in dramatic change in the plant's response to salinity (Shannon, 1997). Although *C. ciliaris* response to salinity stress has been a topic of many researchers (Arshad et al., 2000; Hacker and Waite, 2001; Jorge et al., 2008; Ksiksi and El-Shaigy, 2012); to best of our knowledge, no study has evaluated and characterized the *C. ciliaris* genotypes in terms of agro-morphological attributes and dry matter yield responses so far. This study evaluates the morphological and biomass yield responses of *C. ciliaris* genotypes to water salinity in pot culture trial.

In a study of salt stress on buffelgrass and its effects on productivity decline; Lanza Castelli et al. (2010) has found that *C. ciliaris* accession, Texas 4464, is susceptible to salt stress at 300 mM NaCl concentrations at the seedling stage, while Americana showed tolerance against salinity. The fresh weight, root length, and plant height of these accessions were least affected by salinity. However, screening and selection of large numbers of buffelgrass genotypes for salinity tolerance is lacking. Therefore, the current research was undertaken with the aim to identify superior genotypes for forage production under hot arid conditions of UAE using naturally available low quality saline water. The results of this research are expected to provide useful information that can be used to group accessions for relative comparison and evaluation of biomass yield in areas where problems of soil and water salinity are increasing.

## Materials and methods

### Site description

The field trials were conducted for three growing seasons during 2003–2005 on the experimental farm of the International center for Biosaline Agriculture (ICBA), located on the eastern side of Dubai between 25°5′ N and 55°23′ E with an elevation of 30 m above mean sea level. The soil of the experimental field is Carbonatic, Hyperthermic Typic Torripsamment having a negligible level of inherent soil salinity (0.2 dS m^−1^). The study area is characterized by very hot, dry days in summer (April–October), when temperatures can reach 45°C, while the winter days (December–February) are mainly cooler and dry with low average night-time temperatures (10°C). Because of the aridity, and relatively cloudless skies, there are great extremes of temperature variation, but there are also wide variations between the seasons. Air temperature follows a regular seasonal trend, with a minimum in January and a maximum in July. According to the ICBA weather station data the annual average temperature of the study area was 10°C during the month of January while average maximum temperature during summer, July, was 45°C. The average annual rainfall is < 80 mm and falls in short, torrential bursts during the summer months.

### Plant material, experimental design, and management practices

**Figure 1:**
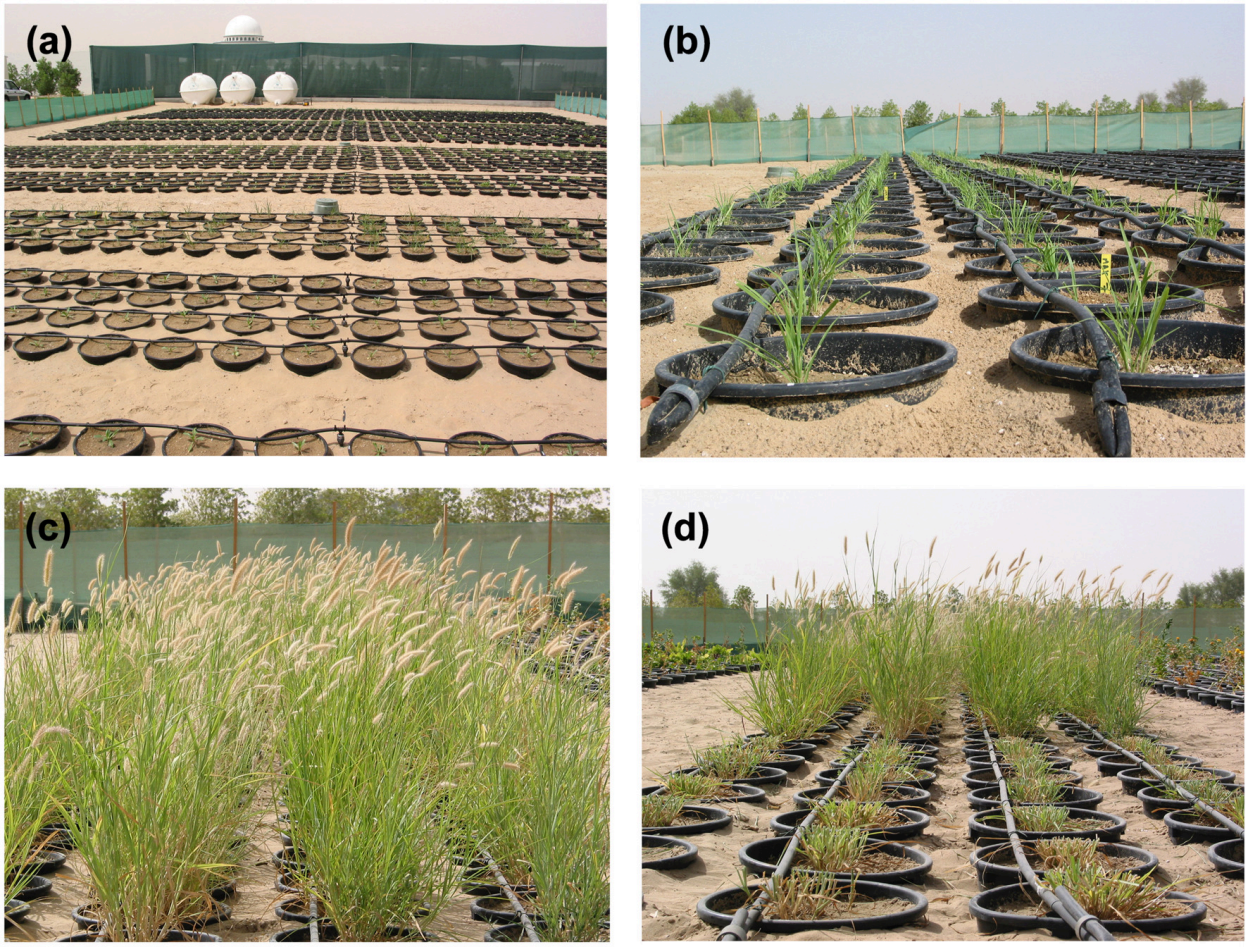
**Screening *Cenchrus ciliaris for salt tolerance in pots buried in open field at Research Station, ICBA, Dubai, UAE*. (A)** Germination, **(B)** growth and tillering, **(C)** heading stage, and **(D)** harvesting.

In total, 160 accessions of buffelgrass (*C. ciliaris* L.) were used in this experiment as shown in Table 1. The seeds of *C. ciliaris* L. were received from United States Department of Agriculture (USDA); originated from Asia, Africa, Australia, India, USA and some local landraces and commercial cultivars were also included in the study. Seeds of *C. ciliaris* genotypes (three per pot) were sown in poly vinyl chloride pots (30 × 30 cm) on 2nd March 2003. The pots were buried in the open field (Figure 1), near the net house at experimental research station (ICBA, Dubai, UAE) for three consecutive years (2003–2005). After uniformity of emergence, seedlings were thinned to one plant per pot to maintain good stand establishment. Experimental soil achieved field capacity and permanent wilting point at 0.1 bar and 15 bars, respectively. All pots were irrigated to upper limit of field capacity before planting.

**Table 1:** **Buffel grass (*Cenchrus ciliaris* L.) germplasm collection with genebank entry numbers and country of origin**.

**S. No**.	**Accessions**	**Species**	**Country of origin**	**S. No**.	**Accessions**	**Species**	**Country of origin**	**S. No**.	**Accessions**	**Species**	**Country of origin**	**S. No**.	**Accessions**	**Species**	**Country of origin**
1	ICBA 22	*C. ciliaris*	UAE	41	PI 299521	*C. ciliaris*	South Africa	81	PI 409263	*C. ciliaris*	South Africa	121	PI 409528	*C. ciliaris*	South Africa
2	ICBA 38	*C. ciliaris*	UAE	42	PI 307619	*C. ciliaris*	Australia	82	PI 409264	*C. ciliaris*	South Africa	122	PI 409556	*C. ciliaris*	South Africa
3	PI 153671	*C. ciliaris*	Kenya	43	PI 308595	*C. ciliaris*	Australia	83	PI 409265	*C. ciliaris*	South Africa	123	PI 409581	*C. ciliaris*	South Africa
4	PI 156546	*C. ciliaris*	Zimbabwe	44	PI 315681	*C. ciliaris*	United States	84	PI 409266	*C. ciliaris*	South Africa	124	PI 409585	*C. ciliaris*	South Africa
5	PI 161631	*C. ciliaris*	South Africa	45	PI 339892	*C. ciliaris*	Australia	85	PI 409267	*C. ciliaris*	South Africa	125	PI 409653	*C. ciliaris*	South Africa
6	PI 161633	*C. ciliaris*	South Africa	46	PI 365650	*C. ciliaris*	Tanzania	86	PI 409270	*C. ciliaris*	South Africa	126	PI 409669	*C. ciliaris*	South Africa
7	PI 161637	*C. ciliaris*	South Africa	47	PI 365651	*C. ciliaris*	Tanzania	87	PI 409272	*C. ciliaris*	South Africa	127	PI 409689	*C. ciliaris*	South Africa
8	PI 185564	*C. ciliaris*	South Africa	48	PI 365720	*C. ciliaris*	Tanzania	88	PI 409273	*C. ciliaris*	South Africa	128	PI 409692	*C. ciliaris*	South Africa
9	PI 193445	*C. ciliaris*	Australia	49	PI 385321	*C. ciliaris*	Tanzania	89	PI 409274	*C. ciliaris*	South Africa	129	PI 409704	*C. ciliaris*	South Africa
10	PI 203363	*C. ciliaris*	South Africa	50	PI 409174	*C. ciliaris*	South Africa	90	PI 409280	*C. ciliaris*	South Africa	130	PI 409718	*C. ciliaris*	South Africa
11	PI 216374	*C. ciliaris*	United States	51	PI 409185	*C. ciliaris*	South Africa	91	PI 409281	*C. ciliaris*	South Africa	131	PI 409720	*C. ciliaris*	South Africa
12	PI 225012	*C. ciliaris*	Ghana	52	PI 409188	*C. ciliaris*	South Africa	92	PI 409282	*C. ciliaris*	South Africa	132	PI 414447	*C. ciliaris*	South Africa
13	PI 225583	*C. ciliaris*	South Africa	53	PI 409194	*C. ciliaris*	South Africa	93	PI 409292	*C. ciliaris*	South Africa	133	PI 414452	*C. ciliaris*	South Africa
14	PI 226090	*C. ciliaris*	Kenya	54	PI 409198	*C. ciliaris*	South Africa	94	PI 409293	*C. ciliaris*	South Africa	134	PI 414499	*C. ciliaris*	South Africa
15	PI 243198	*C. ciliaris*	Zimbabwe	55	PI 409201	*C. ciliaris*	South Africa	95	PI 409295	*C. ciliaris*	South Africa	135	PI 414508	*C. ciliaris*	South Africa
16	PI 243199	*C. ciliaris*	Zimbabwe	56	PI 409205	*C. ciliaris*	South Africa	96	PI 409298	*C. ciliaris*	South Africa	136	PI 414513	*C. ciliaris*	South Africa
17	PI 245375	*C. ciliaris*	India	57	PI 409212	*C. ciliaris*	South Africa	97	PI 409301	*C. ciliaris*	South Africa	137	PI 414521	*C. ciliaris*	South Africa
18	PI 253261	*C. ciliaris*	South Africa	58	PI 409214	*C. ciliaris*	South Africa	98	PI 409302	*C. ciliaris*	South Africa	138	PI 414529	*C. ciliaris*	South Africa
19	PI 253263	*C. ciliaris*	South Africa	59	PI 409216	*C. ciliaris*	South Africa	99	PI 409309	*C. ciliaris*	South Africa	139	PI 442096	*C. ciliaris*	Japan
20	PI 253269	*C. ciliaris*	South Africa	60	PI 409217	*C. ciliaris*	South Africa	100	PI 409327	*C. ciliaris*	South Africa	140	PI 443507	*C. ciliaris*	Mexico
21	PI 271198	*C. ciliaris*	India	61	PI 409221	*C. ciliaris*	South Africa	101	PI 409348	*C. ciliaris*	South Africa	141	PI 516516	*C. ciliaris*	Morocco
22	PI 271203	*C. ciliaris*	India	62	PI 409222	*C. ciliaris*	South Africa	102	PI 409352	*C. ciliaris*	South Africa	142	Grif 1619	*C. ciliaris*	Australia
23	PI 271206	*C. ciliaris*	India	63	PI 409223	*C. ciliaris*	South Africa	103	PI 409361	*C. ciliaris*	South Africa	143	Grif 1621	*C. ciliaris*	Kenya
24	PI 271208	*C. ciliaris*	India	64	PI 409224	*C. ciliaris*	South Africa	104	PI 409365	*C. ciliaris*	South Africa	144	Grif 1622	*C. ciliaris*	Botswana
25	PI 271209	*C. ciliaris*	India	65	PI 409226	*C. ciliaris*	South Africa	105	PI 409367	*C. ciliaris*	South Africa	145	Grif 1637	*C. ciliaris*	Tanzania
26	PI 271214	*C. ciliaris*	India	66	PI 409228	*C. ciliaris*	South Africa	106	PI 409375	*C. ciliaris*	South Africa	146	Grif 1639	*C. ciliaris*	Pakistan
27	PI 271219	*C. ciliaris*	India	67	PI 409229	*C. ciliaris*	South Africa	107	PI 409379	*C. ciliaris*	South Africa	147	Grif 1640	*C. ciliaris*	Australia
28	PI 271593	*C. ciliaris*	India	68	PI 409230	*C. ciliaris*	South Africa	108	PI 409381	*C. ciliaris*	South Africa	148	MAF 74	*C. ciliaris*	UAE
29	PI 275104	*C. ciliaris*	India	69	PI 409231	*C. ciliaris*	South Africa	109	PI 409385	*C. ciliaris*	South Africa	149	ICBA 49	*C. ciliaris*	UAE
30	PI 279596	*C. ciliaris*	Philippines	70	PI 409232	*C. ciliaris*	South Africa	110	PI 409398	*C. ciliaris*	South Africa	150	C MAK 1	*C. ciliaris*	UAE
31	PI 284831	*C. ciliaris*	South Africa	71	PI 409234	*C. ciliaris*	South Africa	111	PI 409399	*C. ciliaris*	South Africa	151	C MAK 2	*C. ciliaris*	UAE
32	PI 284834	*C. ciliaris*	Morocco	72	PI 409235	*C. ciliaris*	South Africa	112	PI 409400	*C. ciliaris*	South Africa	152	C MAK 3	*C. ciliaris*	UAE
33	PI 284835	*C. ciliaris*	India	73	PI 409236	*C. ciliaris*	South Africa	113	PI 409401	*C. ciliaris*	South Africa	153	C MAK 4	*C. ciliaris*	UAE
34	PI 292637	*C. ciliaris*	Argentina	74	PI 409237	*C. ciliaris*	South Africa	114	PI 409402	*C. ciliaris*	South Africa	154	C MAK 5	*C. ciliaris*	UAE
35	PI 293325	*C. ciliaris*	Argentina	75	PI 409238	*C. ciliaris*	South Africa	115	PI 409408	*C. ciliaris*	South Africa	155	C MAK 6	*C. ciliaris*	UAE
36	PI 294595	*C. ciliaris*	Australia	76	PI 409240	*C. ciliaris*	South Africa	116	PI 409426	*C. ciliaris*	South Africa	156	C MAK 7	*C. ciliaris*	UAE
37	PI 295655	*C. ciliaris*	Zimbabwe	77	PI 409241	*C. ciliaris*	South Africa	117	PI 409429	*C. ciliaris*	South Africa	157	C MAK 8	*C. ciliaris*	UAE
38	PI 295659	*C. ciliaris*	Zimbabwe	78	PI 409250	*C. ciliaris*	South Africa	118	PI 409438	*C. ciliaris*	South Africa	158	Gayndah	*C. ciliaris*	AUS
39	PI 298980	*C. ciliaris*	Zimbabwe	79	PI 409254	*C. ciliaris*	South Africa	119	PI 409442	*C. ciliaris*	South Africa	159	Biloela	*C. ciliaris*	AUS
40	PI 299505	*C. ciliaris*	South Africa	80	PI 409262	*C. ciliaris*	South Africa	120	PI 409444	*C. ciliaris*	South Africa	160	Commercial	*C. ciliaris*	USA

The soil was collected from an open area of ICBA that had not been previously irrigated with saline water. Textural analysis showed that the resulting soil had 98% sand, 0.5% silt, and 1% clay, which would be classified as sandy soils. The plastic pots were filled with soil mix (20 kg/pot) containing 50% sand, 25% organic fertilizer, and 25% peat moss. The fertilizer NPK was mixed periodically at the start of the trial and after each harvest. The weeds were eradicated by hand weeding.

### Irrigation and saline water treatment application

The *C. ciliaris* accessions were arranged in the main plot while saline water treatment (0, 10, 15, and 20 dS m^−1^) was randomized in RCBD split plot design. Initially, the pots were irrigated with fresh water up till 1 month to facilitate the germination and seedling establishment. One pot was assigned to each accession per replication and four replications per treatment were maintained throughout the trial. The saline water treatments were applied 1 month after sowing and each salinity treatment was made by diluting ground water (EC: 25 dS m^−1^) with fresh water (1.5 dS m^−1^) in a separate tank to achieve the target salinities and then delivered to the pots via drip irrigation system. Irrigations were applied on daily basis at rates equivalent to ET_0_ plus 20% for leaching requirements. The four salinity levels were maintained constantly throughout the growth period during all the years.

The field experiment was equipped with a drip system (pressure compensating (PC), micro flapper with 4 L hr^−1^ flow rate) and 0.5 m distance between rows and 0.25 m between drippers. Each pot has one dripper; the pot area was 0.12 m^2^ which means that for each 4 liters the depth would be 33 mm hr^−1^. Irrigation monitoring, scheduling and salinity management were achieved using Decagon®; sensors. During the summer season (9 months from March–November) the pots were irrigated 30 min per day which means an irrigation depth of 16 mm per pot with the leaching fraction, but during mild winter season (3 months from December–February) the irrigation duration was 20 min which means an irrigation depth of 11 mm per day with the leaching fraction. The drippers were tested biweekly to check the distribution uniformity and coefficient using the method of the low quarter average divided by the average of the whole readings, the data (not shown) that the distribution uniformity was not < 85% with a distribution coefficient of 80%.

### Harvesting and biomass yield measurements

The plants were harvested at heading stage and weighed with an analytical balance. Table 2 demonstrates the harvesting dates and number of harvests (cuts) obtained during each year. For each harvest, the total fresh weight of the collected biomass samples was weighed in g/pot. The samples were dried in a forced air oven at 60°C for 72 h and dry matter yield (DW) was determined.

**Table 2:** **Sequence of harvesting schedule, stage, cut date, and number of cuts in perennial crop**.

**Harvesting schedule**	**Harvesting stage**	**Cut date**	**Year**	**Number of cuts**
1st cut	Heading stage	06-05-03	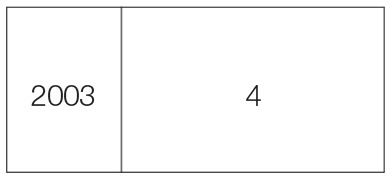	
2nd cut	// //	14-07-03		
3rd cut	// //	29-09-03		
4th cut	// //	15-12-03		
5th cut	// //	01-03-04	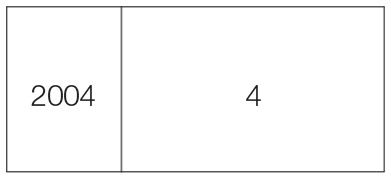	
6th cut	// //	15-06-04		
7th cut	// //	06-09-04		
8th cut	// //	13-12-04		
9th cut	// //	08-03-05	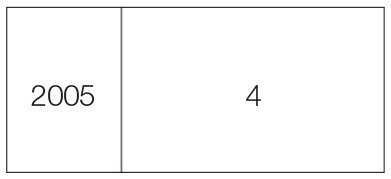	
11th cut	// //	16-06-05		
12th cut	// //	27-08-05		
13th cut	// //	12-12-05		

Cenchrus ciliaris for salinity tolerance comparison trial for year 2003–2005.

### Data and statistical analysis

Statistical analyses were performed on fresh biomass (FW) and dry weight (DW) in two steps:

1. The experimental data for each cut (fresh weight, dry weight) of 160 genotypes were analyzed using a general linear model with salinity treatment and genotypes as fixed factor and year as a random factor using SPSS for Windows version 17.0 (SPSS Inc., Chicago, IL, USA). Difference between treatments means were compared using Tukey's HSD test.
2. Principal component analysis (PCA) was performed on genotypic trait means (FW, DW), to partition the performance of accessions under salt stress conditions. The variate stands for any response variable and “individual” stands for any entry in this analysis. PCA helps the selection of entries, at 25% intensity of selection, based on entries loadings on the first and the second components at each salinity level. The accessions were selected and ability of each entry was determined using its loadings on two components of PCA.
3. Hierarchical cluster analysis of 160 buffelgrass accessions using JMP 12.0 software was carried out taking fresh biomass yield and dry weight taking into account means of all years at overall salinity. The between-groups linkage method of clustering was adopted using Euclidean distances.

## Results

### Impact of salinity on average biomass yield of *C. ciliaris*

**Figure 2:**
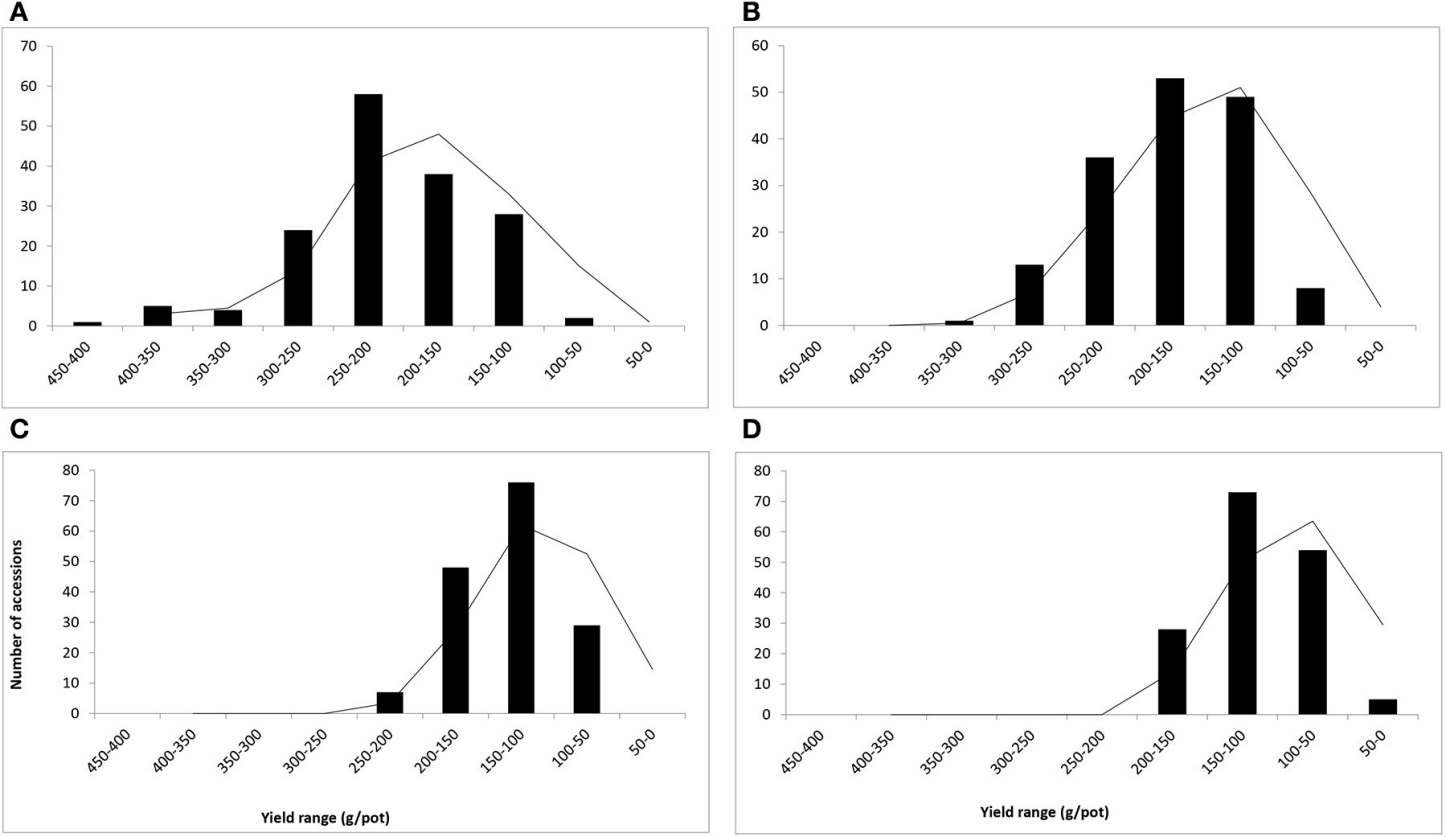
**Frequency distribution and yield range (g/pot) of 160 buffelgrass accessions (Means of fresh weight at A–D at heading stage)**. Whereas, **A** = 0, **B** = 10, **C** = 15, and **D** = 20 dS m^−1^ salinity level.

**Figure 3:**
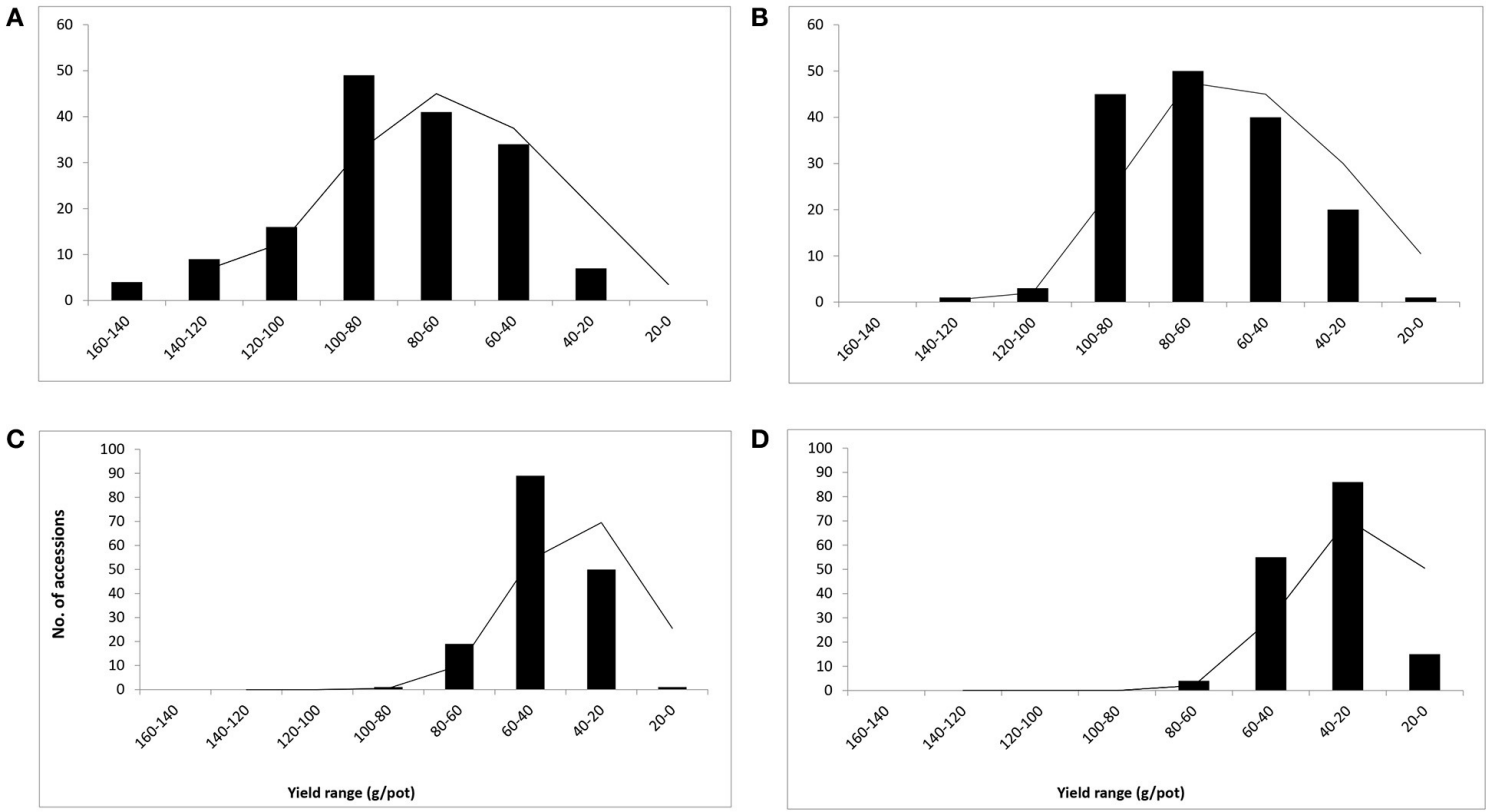
**Frequency distribution and yield range (g/pot) of 160 buffelgrass accessions (Mean of dry weight at A–D at heading stage)**. Whereas, **A** = 0, **B** = 10, **C** = 15, and **D** = 20 dS m^−1^ salinity level.

The salinity treatments significantly influenced the growth and biomass yield of various *C. ciliaris* accessions. The average biomass yield (fresh and dry weight) were calculated by taking the average of 3 years' data for each salinity level (Figures 2, 3). Fresh weight (FW) ranged between 50 and 450 g/pot in control, 50–350 g/pot at low salinity (10 dS m^−1^), 50–300 g/pot at medium high salinity (15 dS m^−1^) and 50–200 g/pot at high salinity (20 dS m^−1^) (Figures 2A–D). The curve was bell shaped in control pots and right skewed distribution when the values clustered more toward lower production groups at high salinity (Figure 2D). Accession 127 proved to be stable and higher yielder that produced the maximum FW (400.8 g/pot) in control and 348.9 g/pot fresh biomass at 10 dS m^−1^. However, at medium and higher salinity (15, 20 dS m^−1^), its biomass yield decreased significantly and produced 180.1 and 122.3 g/pot, respectively. Accession 49 proved to be stable genotype that produced the 2nd highest biomass yield (351.1 g/pot) in the control pots while 292.4 g/pot at 10 dS m^−1^, 219.3 g/pot at 15 dS m^−1^, and 181.7 g/pot at 20 dS m^−1^, respectively. At medium and higher salinity (15 and 20 dS m^−1^), accession 30 produced 233.8 g and 154.2 g/pot FW and demonstrated as medium salt tolerant genotypes.

The dry weight range was in the range of 20–160 g/pot in control, 20–140 g/pot at 10 dS m^−1^, 100–20 g/pot at 15 dS m^−1^, and 20–80 g/pot at 20 dS m^−1^ respectively (Figures 3A–D). The curve distribution was bell shaped in control pots and right skewed at high salinity (20 dS m^−1^) (Figure 3D). The range of variation reduced with an increase in salinity levels and ranged between 20 and 160 g/pot when salinity varies from 0 to 20 dS m^−1^. The reduction in dry biomass was 87.5, 85.7, 80, and 75% at 10, 15, and 20 dS m^−1^, respectively (Figures 3A–D). The average mean DW was 148.9 g/pot recorded for accession 49 in control while accession 127 produced the highest dry biomass (141.4 g/pot), in control pots, respectively (data not shown). The accession 124 produced the maximum DW (80.3 g/pot) at 20 dS m^−1^ salinity and proved to be stable and higher yield genotype. At highest salinity (20 dS m^−1^), accession 49 produced the maximum dry biomass (65.6 g/pot) (data not shown).

### Effect of salinity on total annual yield of *C. ciliaris*

**Figure 4:**
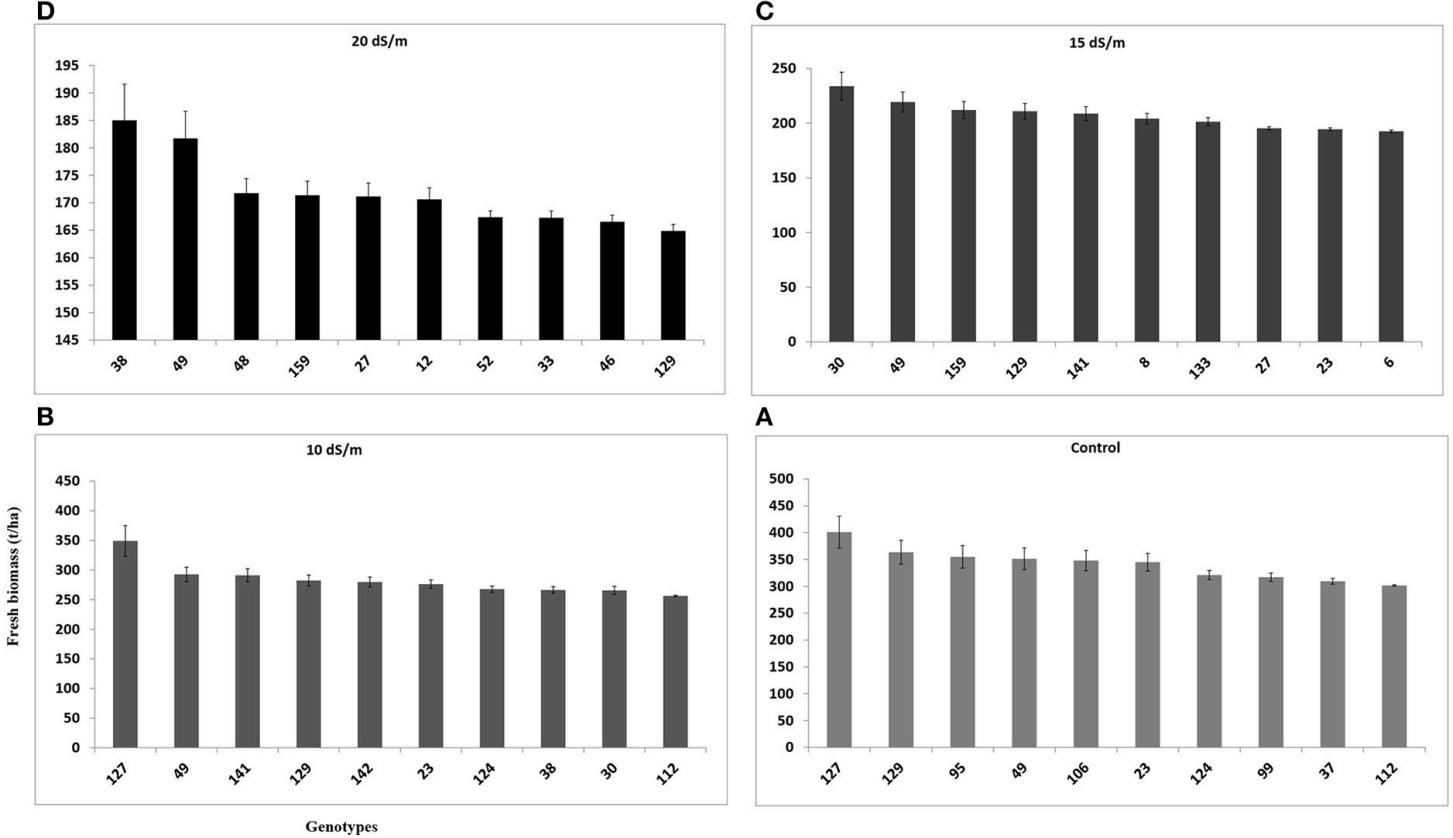
**Effect of different irrigation water salinity levels on fresh biomass yield (total annual yield) of TOP 10 *Cenchrus ciliaris* genotypes**. Error bars represent standard deviations of the mean of four replicates. Whereas, **(A)** = 0, **(B)** = 10, **(C)** = 15, and **(D)** = 20 dS m^−1^ salinity level.

**Figure 5:**
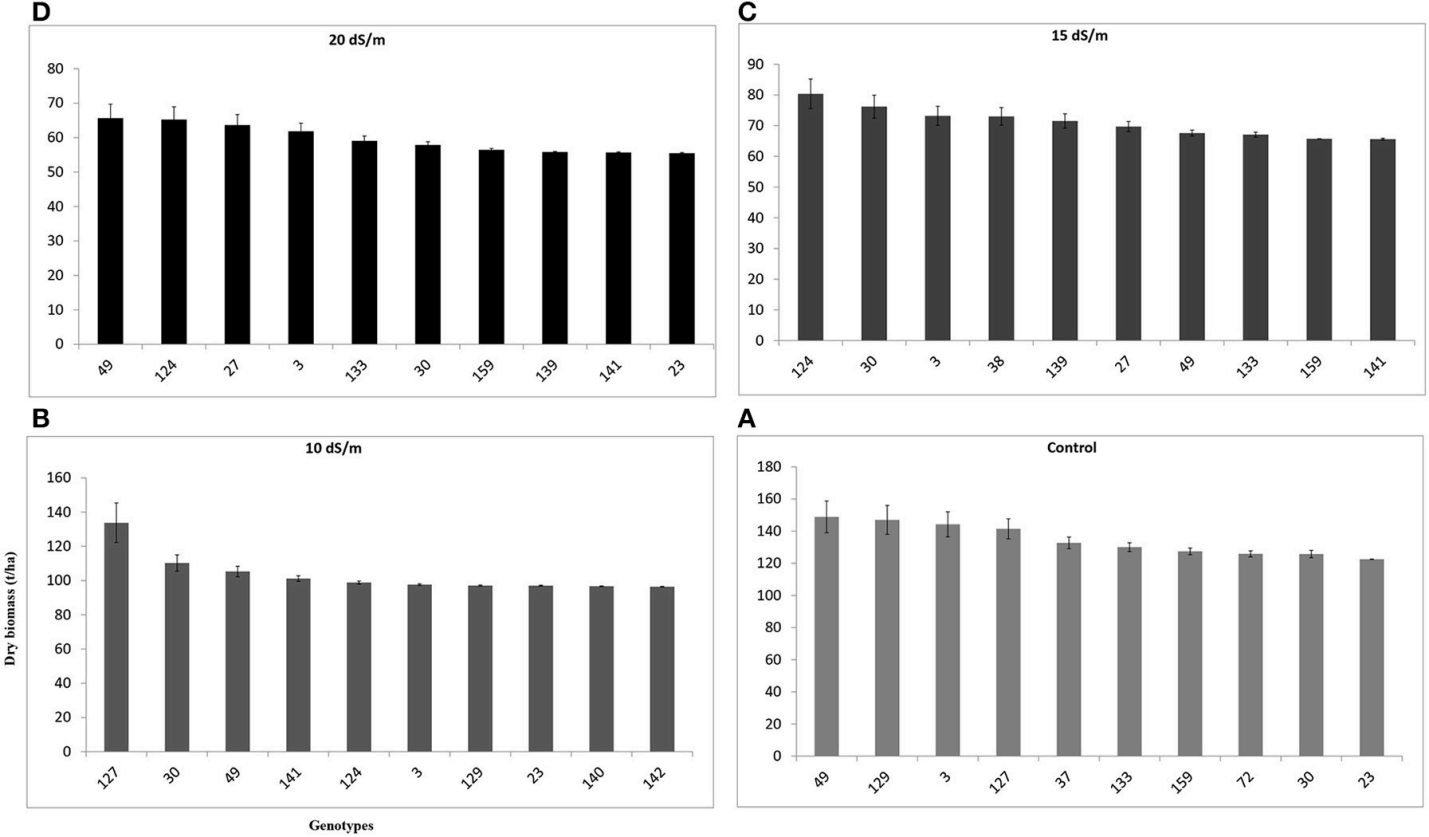
**Effect of different irrigation water salinity levels on dry weight (total annual dry biomass) of TOP 10 *Cenchrus ciliaris* genotypes**. Error bars represent standard deviations of the mean of four replicates. Whereas, **(A)** = 0, **(B)** = 10, **(C)** = 15, and **(D)** = 20 dS m^™1^ salinity level.

Fresh biomass (total annual yield) ranged from 301.6 to 400.8 g/pot in control, 256.2–348.9 g/pot at 10 dSm^−1^, 192.5–233.8 g/pot at 15 dSm^−1^, and 164.8–185.0 g/pot at 20 dS m^−1^) among the top 10 accessions of *C. ciliaris* (Figure 4). The results illustrated that FW in 112, 37, 30, 23, and 46 accessions were more affected by the salinity at 10, 15, and 20 dS m^−1^, respectively. The accessions 49, 159, 129, 30, 38, were the least affected by increasing the salinity level from 0 to 20 dS m^−1^. However, accession 129 produced good fresh biomass yield in control, 10 dS m^−1^, and 15 dS m^−1^ salinity while it was not a stable genotype at higher salinity (20 dS m^−1^) that produced less FW as compared to other top 10 genotypes (Figure 4). The salinity tolerance values in DW from all buffelgrass accessions show a high degree of variability under the various salinity levels. Average annual DW yield ranged from 122.5 to 148.9 g/pot in control; 96.4–133.7 g/pot at 10 dS m^−1^; from 65.6 to 80.3 g/pot at 15 dS m^−1^ and 55.4–65.6 g/pot at higher salinity (20 dS m^−1^). The highest DW (148.9 g/pot) was produced by accession 49 in control and the lowest one (55.4 g/pot) was recorded in accession 23 following treatment at the highest salinity level (20 dS m^−1^) (Figure 5).

### Evaluation of performance of *C. ciliaris* accessions through multivariate analysis

#### Principle component analysis

**Figure 6:**
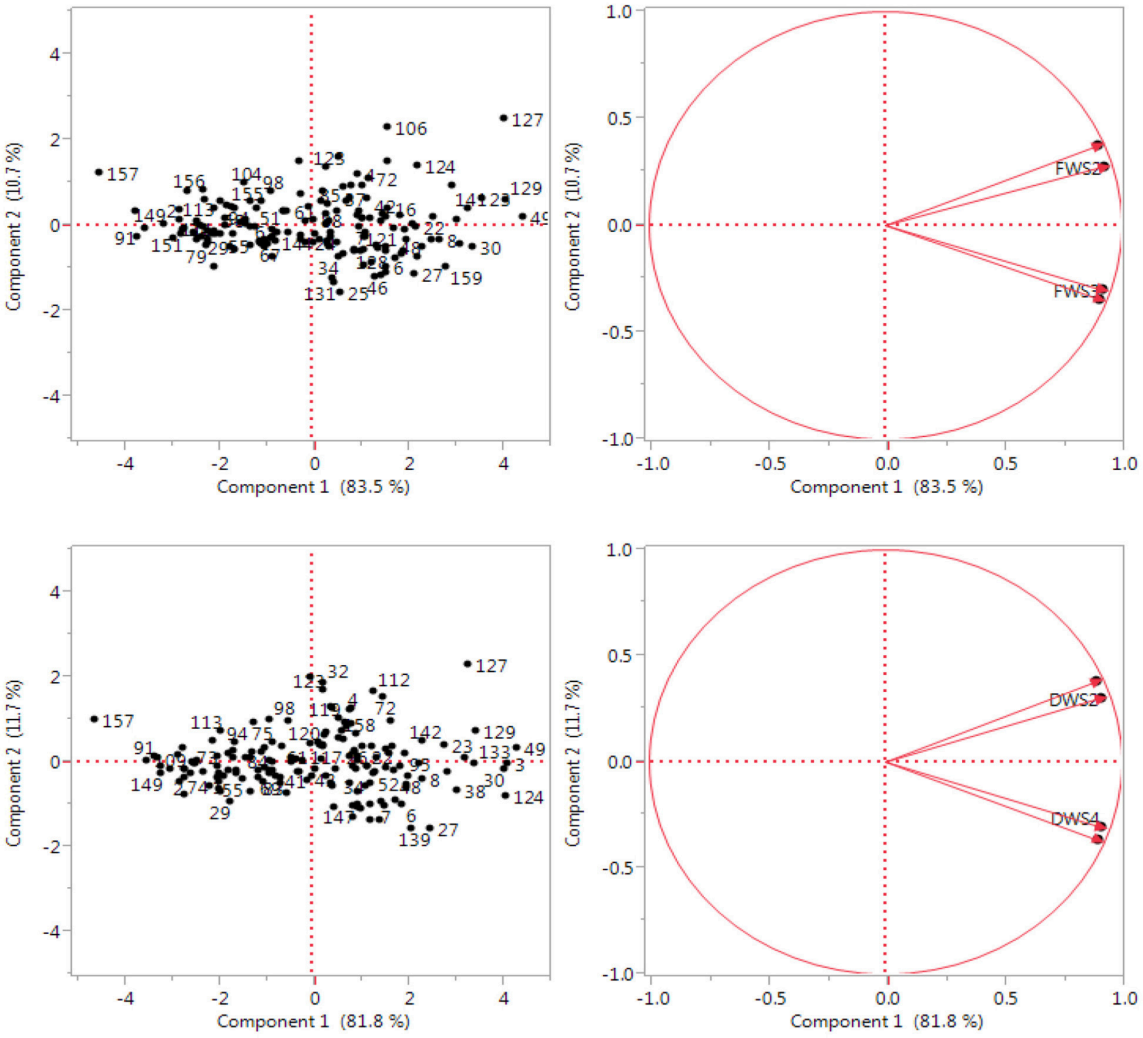
**Principal component analysis of the variables: Fresh weight (FW) and dry weight (DW) at four water salinity levels (0, 10, 15, 20 dS m^−1^)**. Two dimensional scatter plot of genotypic relationship among 160 *Cenchrus ciliaris* accessions as revealed by first two principal components.

The results of principal components analysis show that out of four components, only 1 had extracted Eigen values over 1. This is based on Chatfield and Collin (1980) assumption which stated that components with an Eigen value of < 1 should be eliminated. The extracted component was subsequently rotated according to Varimax rotation in order to make interpretation easier and fundamental significance of extracted component to the irrigation water salinity. Principle component PC 1 was extracted having Eigen value >1 and contributed 83.5% of the total variability among the *C. ciliaris* genotypes assessed for fresh biomass (Figure 6). However, the PC 2 depicted 10.7% of the total variation for the same parameter. Furthermore, PC1 contributed 81.8% of the total variability among the 160 genotypes and PC2 only depicted 11.7% of the total variation for dry weight (DW). Salinity applies a gradual selection pressure on the entries that was higher for dry biomass than for fresh biomass. PCA of *C. ciliaris* genotypes revealed diverse grouping pattern. The separation on the basis of PC1 and PC2 revealed that the genotypes were scattered in all the quarters, which show the high level of genotypic variation among the accessions (Figure 6). The first principal component correlated with five of the original variables (DWS2, FW2, FWS3, DWS1, and FWS4) and more strongly with the DWS2. In fact, we could state that based on this correlation, principal component is primarily a measure of the DWS2 (data not shown). In the first principal components, accessions 49, 22, 16, 42, 8, 149, showed more genotypic variation based on FW and are located close to axis line. In the second principal components, accessions 49, 133, 23, 3, 30, 6, showed more genotypic variation based on dry weight (DW) trait and are also located close to axis line.

#### Hierarchical cluster analysis

**Figure 7:**
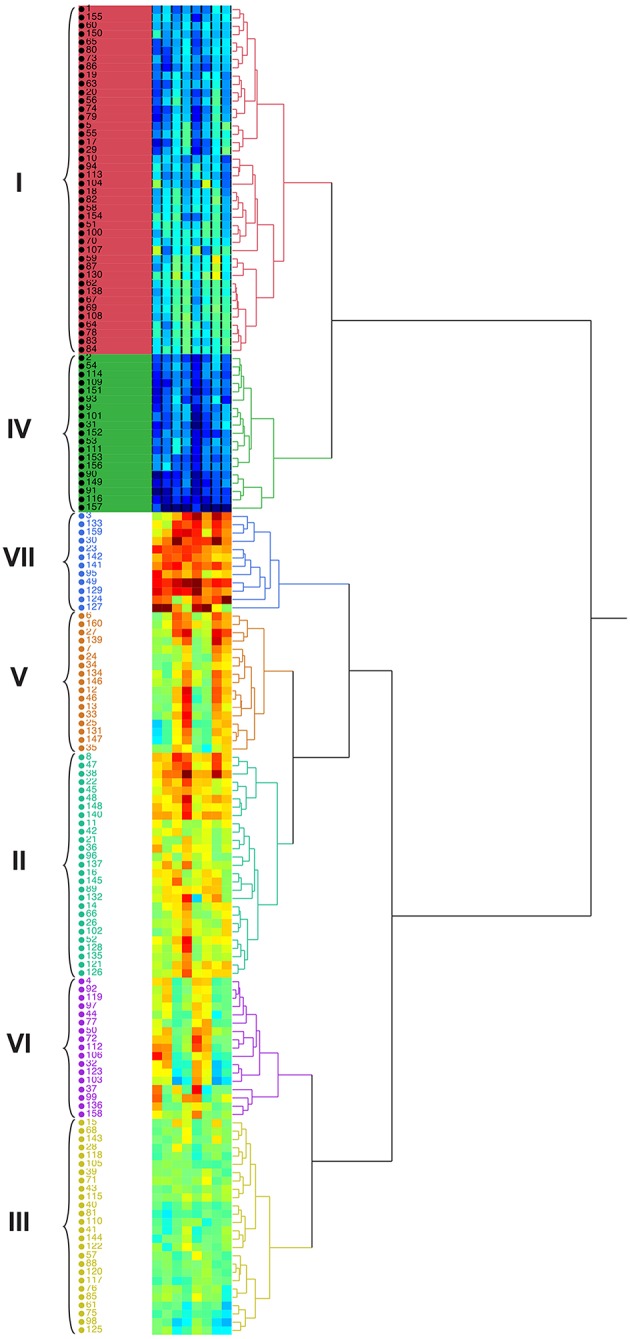
**Dendrogram showing the relationship among 160 accessions of *Cenchrus ciliaris* based on plant biomass in a Hierarchical Clustering analysis**.

Germplasm evaluation for quantitative traits may help to work out the relative importance of various traits within each cluster. Hierarchical cluster analysis based on agronomical traits (fresh and dry biomass) divided the 160 accessions into 6 main clusters. Maximum number of accessions (42) were present in group I, followed group II (27), group III (26), group IV (19), group V (17), group VI (17), and group VII (12) (Figure 7). Hierarchical cluster analysis showed that some of accessions originated from various geographical areas were grouped into the same cluster, while many others fell into different clusters. Group VII consisted of highly salt tolerant genotypes (3, 133, 159, 30, 23, 142, 141, 95, 49, 129, 124, and 127) that acquired maximum biomass yield at all target salinities and also among the top 10 accessions. Grouping pattern indicated that the clusters were heterogeneous with regard to the geographical origin and some entries from different geographic regions were pooled in the same cluster (Figure 7).

#### Performance of commercial cultivars of *C. ciliaris*

The observations of present study were largely consistent with the available descriptions of commercial cultivars. The genotype 159 (Biloela) was salt tolerant, productive and produced higher biomass than other genotypes at different water salinities (0, 10, 15, 20 dS m^−1^). Other commercial genotypes 158 (Gayndah) and 160 did not show the good performance in terms of fresh and dry biomass and were not stable at medium and high salinities (data not shown).

## Discussion

Yield assessment conducted in field trials provides the most reliable information on salt tolerance; however, spatial and temporal variability in soil salinity (Richards, 1983) make it difficult to obtain reproducible information. The advantages of using pots buried in open field are that the saline conditions can be controlled and constantly monitored. Salt affects the growth of plants by limiting the absorption of water and essential nutrients through roots. Salt stress has an immediate effect on cell growth and enlargement, and high concentrations of salts can be extremely toxic (Munns and Tester, 2008; Hussain et al., 2016). In the present study, there was wide variation among *C. ciliaris* genotypes grown under different salinity levels regarding their biomass yield. The fresh and dry weight of all genotypes was decreased with increase in salt stress. However, salt-sensitive genotypes showed more reduction in their biomass as compared to tolerant genotypes. Lanza Castelli et al. (2010) demonstrated a significant reduction (50–70%) in height and shoot fresh weight at 30 dS m^−1^ in Texas and Biloela accessions. In the present study, the accessions 49, 127, 129, 30, 38, were the least affected by increasing salinity. The accession 129 produced good biomass at low salinity; however, it was not a stable genotype at higher salinity. Therefore, it is quite suitable for low and medium saline areas where water salinity falls in the range 5–15 dS m^−1^, respectively. Our results demonstrate that accessions from Africa were more stable, salt tolerant and attained high biomass yield as compared to accessions from other regions. Previously, it was reported that, *C. ciliaris* is a dominant grass along the sandy beaches of Eritrea on soils with salt content of 0.006–0.251% (Hemming, 1961). Skerman and Riveros (1990), demonstrated that it grows well in saline soils; although it is less salt tolerant than *Chloris gayana, Cynodon* spp., and *Panicum antidotale*.

The DW from all *C. ciliaris* accessions shows a high variability under the various salinity levels. The highest DW was produced by accession 49 and the lowest by accession 23. Similar studies were conducted on other grass species such as Bermuda grass (Rodríguez and Miller, 2000), *Catharanthus roseus* (Jaleel et al., 2008) and *C. ciliaris* (Lanza Castelli et al., 2010) and showed that the plant growth, tiller, number of leaves and dry weight decreased under salt stress. Ions of sodium (Na^+^) and chlorine (Cl^−^) (>40 mmol/L) can be toxic to plants because they can create imbalance in plant nutrition due to decreased nutrient uptake and translocation to new shoots (Munns et al., 2000; Tester and Davenport, 2003; Munns and Tester, 2008). Nawazish et al. (2006) found that shoot dry weight was severely affected in the *C. ciliaris* ecotype from Faisalabad, where it decreased from 29.83 to 8.02 g/plant. High salinity modifies plant metabolisms, which results in altered plant morphology; cultivar type, duration, and intensity of stress determine the extent of morphological modification. These results indicate that dry biomass yield can be used as a screening and selection criteria for evaluating the salt tolerance behavior among a large collection of plant accessions. Based on their relative performance (DW) there is a chance of selecting the genotypes within each salinity level. Several accessions from the top 10 selection with high biomass yield potential are of particular interest as forage resource in salt-affected agro-ecosystems for livestock and are highly suitable for arid and semi-arid hot dry regions.

From present results, we found that, PCA grouped accessions together with more morphological similarities; the clusters did not necessarily include all the accessions from the same or nearby sites. Diversity of populations within a geographical origin and similarity of populations beyond geographical limits have also been reported in *C. ciliaris* genotypes (Jorge et al., 2008). Among the good performed accessions, genotypes 127, 129, 49, 23, 27, 16, and 42 attained higher DW. It was also found that variables (genotypes) close to an axis correlate more with that principal component; one may consider that axis is a combination of its neighboring variables. Divergence studies of morphological and agronomical traits using principal component and cluster analysis have also been reported by other researchers (Warwick et al., 2006; Dawood et al., 2009) which were in support of present investigation that both cluster and PCA disclosed complex relationship among the accessions in a more understandable way. The variability among the accessions from diverse origin could be related primarily to their morphological differences and secondly to agronomic use. Accessions evaluated in this work exhibited a reasonable level of diversity for some of the studied traits (FW, DW) of economic significance providing a resource for future crop improvement. For the improvement of *C. ciliaris*, it would be desirable to use diverse accessions with more variability for use in future breeding programs. For use in natural resource management and for soil stabilization, *C. ciliaris* accessions which are shorter, more prostrate and more rhizomatous should be selected, because they would provide better ground cover (IBPGR, 1984).

A cluster analysis was used in this study to facilitate the evaluation of salt tolerance among different genotypes. The major advantage of the utilization of a multivariate analysis in the evaluation for salt tolerance is the allowance of a simultaneous analysis on multiple parameters and increase in the accuracy in the rankings of genotypes. Another advantage is the convenience to rank genotypes when plants are evaluated at different salt levels, e.g., moderate and high salt levels. Cluster analysis proved their validity to establish genotypic diversity within accessions. These findings are supported by Iqbal et al. (2010) who concluded that quantitative characters revealed more reliability than biochemical markers that are specific for particular conditions. Germplasm evaluation for quantitative traits may help to work out the relative importance of various traits within each cluster. Accessions from different geographical regions were pooled in the same cluster. Our results coincide with the classification of 68 populations of *C. ciliaris* where most of the populations were grouped mainly due to their agronomical attributes and not related to their geographic origin (Jorge et al., 2008). Group VII consists of highly salt tolerant genotypes that acquired maximum biomass yield at all target salinities and were also among the top 10 accessions. Grouping was not associated with the geographic distribution; instead accessions were mainly grouped based on their morphological traits (FW, DW). Thus, it cannot be generalized that all the accessions having same origin would always have low diversity among them. For example, in Australia, where *C. ciliaris* is grown extensively, rainfall can occur during the cooler months and most cultivars of *C. ciliaris* respond poorly to these rains owing to the cold temperatures. Accessions originating from cool, dry environments could be promising for these areas through improved performance in spring, owing to a better response to rains during the cooler seasons (Hacker and Waite, 2001). Divergence studies of morphological and agronomical attributes using principal component and cluster analysis have been made by different researchers (Dawood et al., 2009).

A good starting point in selecting suitable accessions for dry areas is to test accessions with good agro-morphological attributes originated from areas with very low rainfall. Several accessions of potential interest were collected from arid areas of South Africa, Zimbabwe, Tanzania and hot arid environments of UAE and Kenya. Salinity tolerance is generally an important parameter when choosing accessions to evaluate, especially within a country, because of the strong correlation between genotype and environment (G × E). A total of 100 accessions, originating from South Africa are found in four of the five clusters, showing that there is not a strong link between environment and morphology, confirming the findings in a study on dry matter and phenology of *C. ciliaris* in Tunisia (Mseddi et al., 2004). Some other studies also classified American and Gayndah in the same group (Pengelly et al., 1992).

The present study was largely consistent with the available descriptions of commercial cultivars. One main reason for discrepancies among experimental findings could be a strong influence of the environment. Within-cultivar high variation in *C. ciliaris* is a well-established fact (Jorge et al., 2008) that may be one of the reasons why commercial cultivars are not consistent in their performance. They further concluded that accessions belonging to identical agro-morphological groups were found from a wide range of environments in sub-Saharan Africa. Inherent genetic variability present in perennial grasses collected from different habitats of Cholistan desert were evaluated and high amount of genotypic variations were recorded among the accessions (Arshad et al., 2000). A genotypic characterization of the collection could provide further evidence to confirm the classification of this study.

## Conclusions

We found significant variation for salt tolerance among a large collection of buffelgrass genotypes representing global diversity, USDA collection, commercial varieties and local landraces. We identified several accessions that were stable and productive at high salinity and other accessions whom were stable but low yielder. Accession 3, 133, 159, 30, 23, 142, 141, 95, 49, 129, 124, and 127 were stable, salt tolerant and produced good dry biomass yield at target salinities during all years. These accessions can be exploited to widen the genetic base of existing *C. ciliaris* accessions against salt tolerance. These accessions hold an appropriate salinity tolerance potential and can be grown to enhance farm productivity in arid and semi-arid areas of the globe. Furthermore; introduction of salt-tolerant perennial species is a promising alternatives to overcome salinity problems in the arid regions. The conservation of fresh water resources and its use for high value purposes, while using low quality saline water can provide both ecological and economic benefits, essential for sustainable agriculture in dry lands.

## Author contributions

MH conducted the experiment, collected data, and drafted the manuscript. AA designed the experiment, followed upon data collection, provided support through the PCA statistical analysis and edited the manuscript.

### Conflict of interest statement

The authors declare that the research was conducted in the absence of any commercial or financial relationships that could be construed as a potential conflict of interest.
